# Interventions for Alcoholics Who Smoke

**Published:** 1996

**Authors:** David B. Abrams, Peter M. Monti, Raymond S. Niaura, Damaris J. Rohsenow, Suzanne M. Colby

**Affiliations:** David B. Abrams, Ph.D., is professor of psychiatry and human behavior at Brown University School of Medicine and director of the Division of Behavioral Medicine at the Miriam Hospital, Providence, Rhode Island. Suzanne M. Colby, Ph.D., is a research scientist in the Center for Alcohol and Addiction Studies, Brown University School of Medicine, Providence, Rhode Island. Peter M. Monti, Ph.D., is Career Research Scientist at the VA Medical Center and professor of psychiatry and human behavior and associate director of the Center for Alcohol and Addiction Studies, Brown University School of Medicine, Providence, Rhode Island. Raymond S. Niaura, Ph.D., is associate professor of psychiatry and human behavior at the Brown University School of Medicine and in the Division of Behavioral and Preventive Medicine at the Miriam Hospital, Providence, Rhode Island. Damaris J. Rohsenow, Ph.D., is research clinical psychologist at the VA Medical Center and professor of community health and of the Center for Alcohol and Addiction Studies, Brown University School of Medicine, Providence, Rhode Island

**Keywords:** smoking, AOD dependence, nicotine, intervention, cessation of AODU (alcohol and other drug use), AODR (alcohol and other drug related) disorder, motivational interviewing, expectancy, treatment method, drug interaction

## Abstract

More than 85 percent of adults with a history of alcohol abuse also smoke, and they may be more addicted to nicotine than are smokers without a history of drinking. Alcoholics who smoke also have higher risks of cancer and cardiovascular disease. Indeed, it has been reported that more alcoholics die from tobacco-related diseases than from disorders related to their alcoholism. The complex interaction that exists between alcoholism recovery and tobacco is discussed. In addition, methods are presented for helping alcoholics to stop smoking, including motivating patients, using innovative interventions, and matching effective interventions to the motivational level of the alcoholic. By better understanding the interaction between alcohol and tobacco, scientists can improve treatment outcome and cost-effectiveness for alcoholics who smoke.

Nearly 14 million Americans abuse alcohol (Grant et al. 1994) and an estimated 100,000 deaths each year stem from alcohol abuse and dependence (National Institute on Alcohol Abuse and Alcoholism [NIAAA] 1993). Likewise, approximately 42 million U.S. citizens smoke (approximately 25 percent of the adult population), an addictive behavior that contributes to more than 430,000 deaths annually and is the leading cause of preventable disease, disability, and excess health care costs in the United States (Centers for Disease Control and Prevention [CDC] 1994). Many people engage in both of these health-damaging behaviors. On the average, more than 85 percent of adults with a history of alcohol abuse smoke, and they may be more addicted to nicotine than are smokers without a history of drinking (Monti et al. 1995). Alcoholics who smoke have higher risks for cancer (e.g., of the mouth and throat) and for cardiovascular disease than do nonsmoking alcoholics (CDC 1994). In fact, some scientists (Hurt et al. 1996) report that more alcoholics die from tobacco-related diseases than from alcoholism. Smoking cessation among alcoholics can benefit both individuals and society by reducing the personal, familial, and economic damage caused by the abuse of both substances (Bobo et al. 1995*a*; Orleans 1993).

Researchers and clinicians who study addictions have developed both behavioral and pharmacological interventions for people addicted to either alcohol or tobacco. For example, skills-training programs teach addicts to refrain from drinking or smoking; the drug naltrexone and nicotine patches or gum can be used to mitigate cravings for alcohol and tobacco, respectively. Treatment researchers, however, have been slow to develop therapies addressing dual addiction to nicotine and alcohol. Therapists need to know whether stopping smoking during or after alcoholism treatment enhances or hinders sobriety, but the dearth of research in this area prevents any conclusions from being drawn. Conducting treatment research with alcoholic smokers is crucial to determining how the two addictions can be better addressed during therapy. Because alcohol and tobacco use so frequently co-occurs, it seems ironic that treatment for alcoholism generally ignores tobacco abuse, and vice versa. Thus, how can these oft-entangled addictions be addressed when most treatment research to date rarely has undertaken both alcoholism treatment and smoking cessation simultaneously?

To help smokers in alcoholism treatment quit smoking, scientists and clinicians first must understand the following: (1) how to reach as many recovered and current alcoholic smokers as possible, (2) the mechanisms involved in alcohol-tobacco interactions, and (3) methods for tailoring treatment to patients (Abrams 1995). This article reviews the small amount of existing research on these topics. Attempts to identify and motivate alcoholic smokers and provide effective therapy for them, as this article describes, favor the development of flexible treatment approaches that have as their goal abstinence from tobacco products, because no “safe,” or harmless, level of smoking exists (CDC 1994).

## Reaching Out to Alcoholics Who Smoke

Tobacco and alcohol are used extensively in combination—a fact that can assist health practitioners in reaching out to alcoholics who smoke. In a random community sample, Hughes (1995) found that 34 percent of heavy smokers reported some past or current alcohol-related problem, compared with less than 10 percent of the general population. These and similar findings have led Hughes (1995) and others to conclude that heavy smoking can be a useful screening indicator of alcohol-related problems in clinical settings such as family practice or primary care. Given the prevalence of smoking among alcoholics (mentioned earlier), it is likely that many alcoholic smokers can be found by asking patients first about smoking and then about alcohol use. In addition, alcoholic smokers often have lifestyles that place them at risk for poor health; for example, smokers tend to be disproportionately more prevalent among lower socioeconomic groups (CDC 1994), maintain poorer diets, and generally are more sedentary than nonsmokers (Abrams et al. 1995). Alcoholic smokers are likely to exhibit the same risk factors. By asking about smoking habits and other lifestyle risk factors, a clinician can better reach and motivate patients. In this way, clinicians can reach most alcoholic smokers through ordinary channels, such as the workplace, community organizations (e.g., Alcoholics Anonymous), and the health care system.

## Motivating Alcoholics to Consider Smoking Cessation

The vast majority of alcoholic smokers initially *are not motivated* to quit smoking when they enter alcoholism treatment—either because they do not believe that their smoking constitutes an immediate health risk or because they (as well as many alcoholism treatment staff) believe that their success at achieving abstinence from alcohol may be compromised by stopping smoking. A strong, clear message to patients that tobacco dependence is a life-threatening addiction may be needed to improve motivation. By expecting patients entering alcoholism treatment to stop smoking and by enforcing such a policy, program staff may, over time, increase alcoholic smokers’ motivation to quit. However, resistance to quitting smoking exists in part because little research has either contradicted patient and staff beliefs that quitting might derail sobriety (no research directly supports the idea) or determined how best to change smoking behaviors among alcoholics. Although most studies examining the interactions between smoking and drinking indicate that voluntary smoking cessation does not undermine recovery, the studies are limited by methodological and other issues (discussed later) (Bobo et al. 1995*a*; Hughes 1995). However, mandatory smoking treatment may be more harmful to recovery (Joseph et al. 1993).

Studies confirm that most smokers in alcoholism treatment are not motivated to quit smoking. Alcoholics were surveyed to determine if they would be willing to give up smoking within 6 months of alcoholism treatment. In one study, 86 percent, and in another study, 94 percent of the alcoholic smokers reported that they were not prepared to quit (for review, see Bobo et al. 1995*a*). Among those in day treatment (i.e., programs in which patients spend their days in a treatment program but their nights at home), 72 percent reported that they were not planning to quit smoking within the next 6 months (Monti et al. 1995).

These attitudes (in addition to biases against smoking-cessation therapy among treatment staff—discussed later) form a substantial barrier to clinicians trying to reduce smoking prevalence among alcoholics. To promote smoking cessation, clinicians must learn how not only to help those people interested in quitting but also to motivate the large number of uninterested alcoholic smokers and generate in them the desire to quit smoking (i.e., take a proactive approach). In a proactive approach to smoking-cessation treatment for alcoholics, the counselors must take the initiative early on in the patients’ treatment and encourage them to evaluate their smoking behavior, rather than wait for the patients to seek help in stopping smoking. In addition, clinicians must be able to match interventions to the individual needs of the diverse range of people who are alcoholic smokers (Abrams et al. in press).

### Motivational Interviewing

One intervention that is both proactive and matched to the patient’s needs is motivational interviewing, which already has been proven successful in persuading alcohol-dependent people to enter alcoholism treatment. The motivational interviewing approach also may help clinicians induce smokers to enter smoking-cessation therapy (Miller and Rollnick 1991). The intervention employs a nonconfrontational approach, wherein the clinician recognizes the patient’s ambivalence toward changing a behavior and aims to alter the lack of motivation until he or she is ready to take action. The clinician is empathetic and encourages the patient to explore problems and misgivings, summarizing the patient’s words and highlighting any motivational statements the patient makes. Motivational interviewing emphasizes the patient’s needs and is directly tailored to his or her level of motivation.

For smokers in current alcoholism treatment, motivational interviewing theoretically should promote the success of future attempts at smoking-cessation treatment even when patients are not willing to consider stopping smoking. In this application, motivational interviewing focuses first on helping patients understand the role that tobacco plays in their lives, then on their inner capacity for change. The patients’ enhanced comprehension often increases their motivation to quit, allowing the motivational interviewing intervention to give way to more directive approaches designed to facilitate decisionmaking and skill building—behaviors that help alcoholic smokers remain abstinent from both tobacco and alcohol.

Studies evaluating motivational interviewing have shown that compared with subjects not in motivational interviewing treatment, the therapy results in reduced alcohol consumption among alcoholics (Baer 1995). However, little research has focused on adapting motivational interviewing techniques for smokers in general or for alcoholic smokers in particular. Some of what alcoholic smokers believe (i.e., their expectancies) about how smoking and drinking affect them, however, may provide clues to the appropriate implementation of interventions such as motivational interviewing during recovery from alcoholism.

### Expectancies

During motivational interviewing intervention for alcohol and substance abuse, the clinician asks questions to learn how the patient expects to feel after taking a certain drug (i.e., the patient’s outcome expectancies) (Bandura 1986). The answers to these questions help the clinician discern the patient’s level of motivation and identify the skills the patient will need to attain abstinence (Monti et al. 1995). For example, alcoholics who believe that alcohol increases pleasant feelings or that it helps them reduce stress, cope with anger, or deal with depression clearly will be less confident that they can live without alcohol than alcoholics who accept that other, less destructive ways exist to cope with problems or enhance pleasant feelings. The same principle is true for alcoholics’ expectancies about smoking (Lichten-stein and Glasgow 1992).

Because smoking also is influenced by expectancies, what questions must a clinician ask to evaluate a smoking alcoholic’s motivation to stop smoking? Some survey data illustrate the importance of understanding that alcoholics’ beliefs about smoking can affect their drinking behavior (e.g., some patients may expect that their urge to smoke is greater when they drink, whereas other patients may expect their smoking urges to be lessened during drinking) (Monti et al. 1995).

The same survey also investigated whether alcoholics believed that they would relapse to drinking if they stopped smoking. About 70 percent of the 116 alcoholics sampled said that during sobriety they expected to smoke more when they had an urge to drink than when the urge subsided. Smoking cessation therefore might be particularly difficult early in recovery for some alcoholics. Many recovering alcoholics (58 percent) reported that they smoke at times to cope with the urge to drink, and approximately 70 to 80 percent said that it would be harder for them to stay sober if they quit smoking during alcohol treatment than at a later time (see Monti et al. 1995). Further, those who said they have smoked to cope with urges to drink were less likely to have taken a drink a month later (Monti et al. 1995). These self-reports, however, should not be interpreted as evidence that stopping smoking would cause these alcoholics to relapse to drinking. It is equally likely that people who use cigarettes to cope with drinking urges would find some other coping mechanism (e.g., eating sweets) if they quit smoking.

What is the period during which smoking cessation therapy might best be accepted by alcoholic patients? One study asked patients about their level of motivation to quit smoking during the first week of alcoholism treatment; only 28 percent of recovering alcoholics said they might consider trying to quit in the next 6 months. One month later, however, more than 50 percent of the recovering alcoholics reported a willingness to consider smoking cessation in the next 6 months (Monti et al. 1995).^1^ Thus, even without any smoking intervention, alcoholics become more willing to consider changing their smoking habits after some abstinence from alcohol. This result has important clinical implications, because it suggests that the period immediately following alcoholism treatment may provide a window of opportunity for interventions to increase motivation for quitting smoking (Miller and Rollnick 1991). Some alcoholics may even be ready to stop smoking before they leave treatment, especially if their therapy continues beyond 30 days (e.g., this is often the case in outpatient follow-up to most inpatient and day treatment programs).

In a recent study, Bobo and colleagues (1995*a*) confirmed that a brief, motivationally tailored, self-help intervention for quitting smoking (i.e., a 15-minute counseling session on quitting) given toward the end of residential treatment was readily accepted by staff and patients—93 percent of the patients approached consented to enroll in the study. Bobo and colleagues’ study was based on the stages of change model developed by Prochaska and Di-Clemente (1983). Like motivational interviewing, the model suggests that smokers entering treatment are at different levels of motivational readiness; these levels are termed precontemplation, contemplation, preparation, action, and maintenance. Different stages require different intervention techniques. Thus, when smoking-cessation therapy is matched to the time at which the alcoholics’ levels of motivation are the highest, it may have a positive effect and help alcoholics quit smoking without interfering with their alcoholism treatment.

## Alcohol-Tobacco Interactions: Perception Versus Reality

Alcoholics’ perceptions about how their addictions to tobacco and alcohol are connected may differ completely from the ways in which smoking and drinking actually interact. If alcohol-tobacco interactions do vary from the expectancies that patients report (discussed earlier), then these interactions will affect how smoking-cessation therapy is administered to alcoholics. For example, if most alcoholics—in contrast to what many believe—do not use smoking to cope with urges to drink, then assisting alcoholics in quitting smoking during alcoholism treatment should not endanger their sobriety. In addition, a clinician may find it a simpler task to change an alcoholic’s *perception* of his or her need to continue smoking than to attempt to stop the smoking if the patient actually depends on using tobacco to avoid drinking alcohol.

Researchers have designed several studies to investigate the relationship between smoking and urges to drink. Scientists investigating the effect of tobacco dependence in high-risk drinking situations found that alcoholic smokers with greater tobacco dependence experienced stronger urges to drink and more difficulty and anxiety in role plays of high-risk-for-drinking situations than did alcoholic smokers with less tobacco dependence (Abrams et al. 1995). This result suggests that alcoholics with high levels of tobacco dependence and those who continued to smoke *could* have an increased risk for relapsing to alcoholism. Because the study was correlational, however, the possibility that higher dependence on nicotine is a risk factor for alcohol relapse needs to be confirmed in a prospective study. The subjects’ levels of tobacco dependence *did not* in fact predict the amount of alcohol they were using 6 months after treatment (see Monti et al. 1995). Likewise, some researchers have reported that severity of alcohol dependence and exposure to alcohol cues (e.g., the smell of alcohol) produce greater urges to smoke but that the reverse is not true: Smoking rate and dependence are unrelated to urges to drink.

Nicotine dependence therefore shows no consistent effect on actual drinking outcomes for alcoholics following alcohol treatment (Monti et al. 1995). However, at least four research groups have reported better drinking outcomes among alcoholics who quit smoking than among those who do not (Bobo et al. 1995*a*). Another study also has signaled that alcohol-tobacco interactions do not necessarily make quitting smoking more difficult. One study reported that compared with nonalcoholics, smokers with a history of alcoholism did not have greater smoking withdrawal symptoms after they quit (Hughes 1995) (for further details on smoking and alcohol interactions, see the article by Shiffman, pp. 107–110).

Although too few controlled studies have been performed to date to draw conclusions, the available evidence in this section suggests that alcoholics should be encouraged to stop smoking as soon as possible after they enter treatment for alcohol abuse without risking complications. On the other hand, studies of patients’ expectancies and motivations (reviewed earlier) and the potential harm of mandatory smoking treatment indicate that a guarded approach to introducing smoking cessation therapy during alcoholism treatment should be taken. Thus, neither line of research has demonstrated a clear impact—either positive or negative—of smoking cessation therapy on abstinence from alcohol.

## Treatment Considerations

Once alcoholics begin to consider stopping smoking, whether it is early in alcoholism treatment, at the conclusion of a 30-day residential program, or later—after several months or even years of abstinence from alcohol—special treatments must be tailored to the patients if they are to stop smoking. For example, clinicians must modify existing smoking interventions to the language and symbols that are compatible with substance abuse treatment settings, such as 12-step programs for smoking (Monti et al. 1995).

Treatments also must be cost-effective and easy to deliver to community alcohol- and drug-abuse treatment settings (i.e., they must be generalizable). Other parameters that must be considered when choosing programs for alcoholic smokers include the following: (1) intensity of tobacco cessation treatment, whether minimal (i.e., including self-help programs), moderate (i.e., including interventions such as brief physician advice), or maximal (i.e., involving the use of a formal clinic with specialists); and (2) the content of the program and the modes, methods, and lengths of treatment. For example, treatment options might range from videotapes and books to behavior therapy plus pharmacotherapy, such as nicotine replacement therapy (i.e., nicotine transdermal patches or gum) (Hurt et al. 1995; Abrams et al. 1995). Scientists and clinicians have only begun to determine the most effective of these combinations of factors for alcoholic smokers.

### Studies of Smoking Cessation Treatment for Alcoholics

The studies that have investigated the effects of smoking treatment for alcoholics provide few definitive results. In addition, the most effective treatments for smokers that currently exist (e.g., combined formal behavior therapy and nicotine replacement) have not been tested in a randomized trial^2^ of alcoholic smokers (see Hughes 1995). However, among the few studies that have been performed, one study that offered smoking treatment to a small group of alcoholic smokers (all subjects were male veterans) in a residential alcohol treatment program found that patients who were offered smoking treatment were more likely to continue in residential alcoholism treatment and that 33 percent of them did quit smoking. These results, although not sustained at followup 3 and 6 months later, suggest a positive role for smoking-cessation therapy. Other scientists, however, noted problems, such as failure to adhere to therapy requirements and poorer outcome, when they attempted a mandatory smoking-cessation treatment in hospitalized alcoholics (see Monti et al. 1995).

In one of the only randomized controlled trials conducted to date, Calfas and Martin (1994) recruited volunteers from the Alcoholics Anonymous community who had been abstinent from alcohol for at least 3 months (average sobriety was 4.2 years) and who were presumably highly motivated to stop smoking. Results at 12-months’ follow-up after an intervention revealed that 27 percent of the recovering alcoholics had stopped smoking. Because alcoholic smokers generally are less likely to quit smoking than nonalcoholic smokers, the 27-percent rate of quitting after a smoking intervention is encouraging. Moreover, the participants did not report that their sobriety was compromised by the smoking intervention.

Another study provided smoking treatment to volunteers undergoing in-patient addictions treatment and compared their results with those of people who volunteered for a control group (Hurt et al. 1995). At 1-year followup, 12 percent of the treated smokers but none of the untreated smokers were abstinent from cigarettes. In addition, the treated group showed no increased relapse to drinking. The study noted several methodological problems, including the use of historical controls (i.e., a control group formed from past records of patients similar to those participating in the study) and a sampling bias resulting from nonrandomization, so that the treatment group was weighted with those patients most motivated to quit smoking (Monti et al. 1995). Bobo’s (1995*a*) brief intervention (discussed earlier)—although it was well accepted—resulted in no differences between a control group and an experimental group in smoking cessation. This outcome occurred in part because the treatment was brief; the followup was short; and, as a pilot study, the sample size was small.

Several tentative conclusions may be drawn from the few studies available:

- Voluntary smoking-cessation treatment may be less disruptive for alcoholic patients than mandatory treatment.
- Voluntary cessation or reduction in smoking does not appear to have a deleterious effect on sobriety regardless of the timing of the treatment.
- The more intensive behavioral treatments, as well as nicotine replacement therapy, seem to produce rates of cessation for alcoholic smokers comparable with those of nonalcoholic smokers, but these formal treatments rarely are used for smoking-cessation treatment for alcoholics.
- Studies are hard to interpret, because the small number of subjects who usually volunteer for cessation treatment are likely to be highly motivated to quit.
- Many studies had research design or other methodological limitations. For example, some studies lacked control groups or provided only small amounts of behavioral treatment. Most studies had short followups and small sample sizes.

More definitive research is urgently needed to fill the gaps in our current knowledge and to provide new information that will improve treatment outcomes and cost-effectiveness for alcoholic smokers in the future.

### Transdermal Nicotine Patch and Physician Counseling in Outpatient Substance Abuse Settings

Some addiction researchers have begun to explore whether specific therapies that are effective for nonalcoholic smokers also work for alcoholic smokers. Treatment of moderate intensity, especially with transdermal nicotine (TN) patches, may be a promising intervention for alcoholic smokers (Hughes 1994, 1995; Lichtenstein and Glasgow 1992). For example, scientists have reported beneficial effects of nicotine replacement for smokers with a history of alcohol and drug abuse (Hughes 1995). Physician-delivered smoking-cessation interventions (with a general population of smokers), even when brief, also are known to be effective and can significantly increase smoking abstinence rates for nonalcoholic smokers (Silagy et al. 1994). TN significantly reduces withdrawal symptoms and increases the likelihood of successful smoking cessation (Silagy et al. 1994; Tang et al. 1994). The TN patch, in conjunction with brief counseling and followup, is arguably the most cost-effective smoking intervention available today (Lichtenstein and Glasgow 1992). With proper attention paid to preparing, educating, and training staff, these kinds of smoking interventions probably can be delivered effectively in substance abuse treatment settings (Bobo et al. 1995*a*,*b*). A clear need exists for research to evaluate the effectiveness of these state-of-the-art treatments for alcoholic smokers.

### Tailoring Intervention to Substance Abuse Programs and Organizational Contexts

For smoking cessation treatments of any kind to be effective among alcoholics, clinicians running alcoholism programs must be trained to administer smoking therapies. In addition, strong support from the treatment organization (e.g., the sponsoring agency) and endorsement by all levels of the treatment program’s leadership are essential for the therapy’s success. Some alcohol program counselors and their leaders may themselves still believe that smoking cessation undermines sobriety (Bobo and Davis 1993); positive staff attitudes are critical to the successful implementation of a smoking-cessation intervention (Bobo et al. 1995*a*). Particular attention should be given to the many alcohol counselors who themselves are smokers. Leadership support and a clear smoking policy will help clarify expectations for staff and overcome barriers, such as the myths about drinking and smoking cessation. Physician, counselor, and staff attitudes and behaviors are especially important in outpatient alcohol and substance abuse treatment settings and can strongly influence recruitment, motivation to quit smoking, and actual cessation among alcoholics. Therefore, smoking interventions for outpatient alcoholism settings must be carefully tested for acceptance by both the staff and patients. Staff attitudes can change for the better when smoking-cessation treatment is brought into an alcoholism treatment unit (Hurt et al. 1995).

To minimize encumbrances on alcoholism treatment staff, programs could apply techniques designed to enhance smoking cessation outside treatment settings. For example, smoking-cessation research has demonstrated that physician training, pharmacological aids, followup visits, telephone contacts, and supplemental educational materials can enhance the effectiveness of smoking interventions at reasonable cost and with minimal additional burden on clinic or office staff (Kottke et al. 1988; Manley et al. 1992; Ockene et al. 1994). Counseling by other health care professionals, such as primary care physicians, who typically have the most contact with patients over time, may be especially effective. Patients who received such counseling increased their cessation rates significantly compared with patients who received only brief physician advice (Hollis et al. 1993).

## Conclusions and Future Directions

The ubiquitous nature of smoking among alcoholics has induced researchers to begin unraveling the complex interactions between these two addictions in an effort to develop effective ways of treating each addiction in the context of the other. More treatment research with alcoholic smokers will be useful for several reasons:

- Alcoholic smokers are at high risk for premature death, chronic disease, or disability from smoking and alcoholism combined. Scientists must determine how best to reach the vast majority of alcoholic smokers, motivate them to stop smoking, and ensure that cessation therapy has no ill effects on sobriety.
- Interventions must be developed explicitly for alcoholic smokers and then integrated into substance abuse treatment settings. Research must be conducted to examine risk factors and individual differences in alcohol-tobacco interactions in an effort to advance knowledge of basic mechanisms and to provide data on the need for patient-treatment matching approaches.
- Researchers must bridge the gap between individual (i.e., clinical) research and population (i.e., public health) dissemination research. Clinical research, although valuable, generally focuses on a minority of highly motivated volunteers who do not represent the majority of alcoholic smokers in need of interventions. A public health benefit can be realized if research is balanced in its emphasis on clinical and population dissemination efforts, which apply the findings of clinical studies to a broader population.
- Currently in the United States, pressing health care service delivery and policy questions have been stimulated by escalating health care costs. Future research findings must be incorporated into practice guidelines to help maintain a high quality of care and access for all citizens at reasonable costs. Specifically, the costs of failing to treat smoking among alcoholics must be calculated against the cost of treatment, both in financial and quality-of-life terms and for short- and long-term benefits to society.

An overall approach to reducing smoking prevalence in alcoholics must consider how to achieve the following objectives:

- Proactively reach most of the less motivated alcoholics and accelerate their desire to quit smoking
- Provide effective interventions that can be readily transferred from research to community substance abuse treatment settings
- Deliver a wide range of treatments at reasonable costs
- Develop a strategy to screen and match certain high-risk patients to effective and more specialized treatments.

Additional considerations for maximizing the interventions’ impact include the use of proactive contact with the patient at the point of service; providing personalized approaches and a respectful, sensitive, caring relationship; planning for followup contact; and using materials with information specifically tailored to the unique needs of the alcoholic smoker, such as how to resist cravings to smoke when tempted to drink, so that relapse to both addictions is resisted successfully.

In general, substance abusers are vulnerable to receiving inadequate care, particularly regarding tobacco dependence. However, smoking is the leading preventable cause of premature death, disability, and excess health costs in the United States. Alcoholic smokers as a group are long overdue to receive better treatment for all their addictive behaviors, including their addiction to nicotine. The excess cost to society of alcohol and tobacco abuse is enormous and far surpasses that of AIDS, suicide, and traffic fatalities combined (Abrams et al. 1995). More recovered alcoholics die of tobacco-related disease than of disorders related to their alcoholism (Hurt et al. 1996). Research on the interaction between alcohol and tobacco can contribute significantly to reducing morbidity, mortality, and health costs and to improving the overall quality of life for all people.

## Footnotes

1 Because both the 28 percent early in treatment and the 50 percent later on expressed a willingness to consider quitting in the future (i.e., within 6 months), this result does not contradict the observation that most alcoholics in treatment are not ready to quit during the early stages of treatment for alcoholism.

2 A randomized controlled trial is considered the best study design for research with humans because it eliminates sources of bias. Patients in these studies are randomly assigned to treatment and control groups (instead of being allowed to volunteer for one group or the other).
